# Advice for Health Care Professionals and Users: An Evaluation of Websites for Perinatal Anxiety

**DOI:** 10.2196/11464

**Published:** 2018-12-20

**Authors:** Donna Moore, Virginia Harrison

**Affiliations:** 1 School of Psychology Open University Milton Keynes United Kingdom

**Keywords:** anxiety, female, internet, perinatal, postpartum

## Abstract

**Background:**

Many websites are available with information and resources for perinatal anxiety; however, there is limited research on the quality and content of these sites.

**Objective:**

This study aims to identify what sites are available on perinatal anxiety, identify any information and therapeutic advice given, and review its accuracy and website design.

**Methods:**

We conducted an evaluation of websites for perinatal anxiety. Eligible websites (N=50) were evaluated for accuracy of information, resources for mothers, website quality, and readability.

**Results:**

Information was often incomplete and focused on symptoms rather than risk factors or impact of untreated perinatal anxiety. Websites often had information on treatment (46/50, 92%), but much less on screening (19/50, 38%). Most sites provided at least some resources to support mothers (49/50, 98%), but active, guided support was infrequent (25/50, 50%). Website quality was extremely variable and mostly difficult to read (42/50, 84%).

**Conclusions:**

This study recommends the top 4 websites on perinatal anxiety for health care professionals and users. There is a need for websites to be developed that provide accurate, evidence-based information that women can relate to with quality support resources. Furthermore, these sites should be easy to use and readable.

## Introduction

The perinatal period (ie, including pregnancy and the 12 months following childbirth) marks a profound change and transition for women, which can often be experienced as stressful and overwhelming. Indeed, research in this area suggests that approximately 15%-25% of women experience marked levels of anxiety in this period [1]. Despite being treatable once recognized, most women experiencing perinatal anxiety (PNA) do not seek help for their symptoms [2].

There are a number of reasons why women with PNA may not seek help. While there is mass public awareness about postnatal depression (PND), knowledge about other aspects of perinatal mental health is lacking at both public and health care professional (HCP) levels [3,4]. Thus, symptoms may go unrecognized. For those who do recognize their symptoms, concerns about being regarded as a bad mother [5] and the perceived stigma attached to mental health issues in this period may mean women are less likely to seek treatment [6,7]. This is problematic, as untreated PNA may be associated with a variety of negative outcomes in both the mother and infant, including preterm delivery, low birth weight, PND, excessive infant crying, bonding issues, problematic feeding behaviors, and adverse developmental and mental health problems in children [1,8-10].

One of the challenges in raising awareness of PNA is that the concept is relatively new. Furthermore, PNA has been conceptualized and operationalized in a range of different ways in the literature, varying from self-reported pregnancy-related anxiety symptoms to the exploration of incidence rates of clinically diagnosable anxiety disorders in the antenatal and postnatal periods. Thus, some caution needs to be taken when exploring this construct. For example, it is important not to overpathologize anxiety experienced in this period, as perinatal worry is common and often “normal.” Thus, a distinction needs to be drawn between common transient anxieties often experienced around childbirth and more persistent symptoms that may be more indicative of an anxiety disorder. However, as evidence suggests, even subsyndromal anxiety symptoms may have a negative impact on maternal and infant outcomes [11,12]; thus, support for women who experience subthreshold symptoms remains important.

In addition, there may be a need to further consider how clinically significant PNA is diagnosed and recognized. While higher rates of anxiety disorders, similar to those seen in the general population (including generalized anxiety disorder and obsessive-compulsive disorder), are evident around childbirth [13], a significant proportion of women who experience anxiety in the perinatal period do not meet the criteria for Diagnostic and Statistical Manual of Mental Disorders, Fifth Edition (DSM-V) diagnosis, instead presenting with distressing levels of “maternally focused worry” [14,15]. Therefore, PNA may constitute a clinically distinct phenomenon that is not fully captured by traditional diagnostic methods and scales, resulting in the poorer recognition of these context-specific symptoms. Furthermore, the most common perinatal screening tool currently used in perinatal primary care (the Edinburgh Postnatal Depression Scale; EPDS [16]) was primarily designed to detect PND, which may contribute to the poorer recognition of PNA by both the public and HCPs.

One way to potentially increase public (and professional) awareness of PNA is to use Web-based technology to deliver clinically helpful information, diagnostic and self-help strategies, and evidence-based treatments over the Web. Web-based methods of delivery may be particularly helpful to women in the perinatal period, who may not have the flexibility or time to attend face-to-face appointments with HCPs; and the anonymity the internet affords may circumvent issues associated with stigma [17]. In addition, there is evidence that users who frequently search for health information online tend to be women [18]. Thus, the internet has the potential to break down barriers to help-seeking behaviors in the perinatal period and empower women to self-diagnose and gain support [19,20]. Moreover, the internet can offer HCPs an opportunity to learn more about PNA, delivering up-to-date information about symptoms, risks and outcomes, as well as evidence-based screening and treatment options. Despite this, little is known about the current state of Web-based support and information available for PNA. Thus, women and HCPs need the means to identify which websites are most reliable and provide quality information and resources for PNA.

The literature on perinatal mental health websites is limited. There have been 2 extensive reviews of websites for PND. The first study rated the content and technology of 34 PND-related websites using a general measure of depression, a measure of the website quality and readability [21]. Websites rarely presented current and accurate information on depression, had technological shortcomings, and were difficult to read. A significant number presented misleading information, and some advocated alternative treatment over treatment from an HCP. The second review rated 114 websites using more detailed scales that specifically evaluated information on PND and identified online support resources for women with PND [22]. Findings revealed that the information provided was inadequate and the website quality was variable. While resources for women were often provided, they had limited availability and scope. To date, there is no known review of PNA websites.

Using a similar review method to that of Moore and Ayers [22], this study aims to identify and evaluate current websites for PNA and evaluate their accuracy and quality on a variety of dimensions. This study had 4 key aims: (1) to identify what sites women searching for information about PNA might find; (2) to identify any information and therapeutic advice given and its accuracy; (3) to evaluate website design in terms of navigation, readability, presentation, and accessibility; and (4) to suggest sites that might be most helpful for HCPs and their clients or patients.

## Methods

### Search Strategy

We used lay search terms (Textbox 1) to identify websites an individual looking for information on PNA might find. Thirty combinations of these terms were entered into UK versions of the 4 most popular search engines (Google, Bing, Yahoo, and Ask) in February 2018 [23]. Browser history and cookies were deleted before conducting each search.

As Web users rarely access sites after the first 20 results [24], the first 20 results and featured sites (those paid to appear at the top of the search list) were assessed for inclusion. To be included in the study, websites had to contain at least 500 words about PNA.

Websites that did not contain any information on PNA and that solely focused on any of the following were excluded: other perinatal mental illnesses (eg, PND and puerperal psychosis), stillbirth, bipolar disorder, infant death, abortion, miscarriage, general and childhood anxiety, and general mental health. News items, magazines, blogs, forums, Facebook groups, and other social media were excluded, as were PDFs, videos, scholarly papers, training courses, paid for online therapy, sales promotions, other search engine results and broken links.

**Search terms used to identify websites.**

Antenatal; prenatal; postnatal; perinatal; pregnancy; maternal; mother; baby

+

Anxiety; stress; worry; support; help

### Website Evaluation

There is currently no validated measure that can be used to evaluate websites specific to PNA. While some validated measures exist to assess the quality of generic health and treatment information (such as DISCERN), they are not designed to assess the *accuracy* of information presented or *evaluate* whether it is evidence-based [25]. Therefore, the authors sought to develop a measure that could assess these dimensions, specifically in terms of PNA. As such, the authors developed a rating scale using a modified version of the measure devised by Moore and Ayers [22] to assess PND sites. This included the following 6 sections:

#### Accuracy of Information About Perinatal Anxiety

To assess the accuracy of the information presented on the sites, a review of previously validated scales investigating anxiety in the pre- and postnatal period (including pregnancy-related anxiety) was conducted. Distinct symptoms identified from these scales were compiled to form part of this scale, alongside the DSM-V [26] criteria for anxiety disorders. Similarly, a pragmatic review of the PNA literature was carried out to identify previously published, peer-reviewed papers that identified the risk factors associated with PNA, and its impact on mother and child. Each risk factor and impact outcome were also collated. This resulted in the production of 3 subscales as follows:

##### Symptoms

This subscale examined whether accurate and appropriate information was given about common anxiety symptoms experienced by women in the perinatal period. A checklist of possible PNA symptoms was created by combing those outlined in the DSM-V [26] criteria for anxiety disorders (n=11), and distinct symptoms that were extracted from 3 validated PNA-specific measures (n=8)—The Perinatal Anxiety Screening Scale (PASS) [27], The Postpartum Specific Anxiety Scale (PSAS) [28], and the revised Pregnancy-Related Anxiety Questionnaire-Revised (PRAQ-R) [14]. Websites scored 1 point per symptom listed, with a range of 0-19.

##### Risk Factors

The second subscale included items related to factors that research has found to be linked to PNA, including poor social support, previous mental illness, and previous traumatic life event [10,29]. Again, websites scored 1 point for each risk factor given, with a range of 0-11.

##### Impact

The third subscale was divided into 2 sections (impact on the mother and impact on the infant) and included items research has identified as potential consequences of PNA, including diminished responsiveness to infant cues, low birthweight, and adverse developmental issues [30-32]. Using a similar method to those above, the range of scores was 0-13 (0-8 for maternal impact and 0-5 for infant impact factors). Impact on the father and partner of the mother were excluded, as there was insufficient evidence-based literature. A total score for information accuracy was created by summing symptoms, risk factors, and impact scores, with a possible range of 0-43.

#### Inclusion of Appropriate Treatment and Screening Information

This section noted whether websites included accurate information about appropriate screening tools for PNA and its treatment. In terms of treatment, a meta-review of systematic reviews assessing the treatment efficacy for PNA was carried out and efficacious treatment options were identified. These included face-to-face, group, and online CBT-based treatments, mindfulness and pharmacological interventions such as selective serotonin reuptake inhibitors[33,34]. Each website was categorized as either providing information about evidence-based treatment options, containing no treatment information, or including inaccurate or unsafe information that advocated alternative treatments over formal medical or psychological help. If a site contained information about treatment options, the nature of these treatments was noted.

Websites were also categorized according to whether they (1) provided PNA screening information to users using established, validated screening tools or (2) provided no information at all. They were then further coded according to the nature of the tool along 3 dimensions—generic anxiety scales, PNA-specific scale, PND scale, and the tools themselves were noted.

#### Available Help

Websites also received a score for the number of resources they offered; these were grouped into 3 categories as follows:

##### Tools for Mothers

These encompassed self-directed information and tools, including help-seeking advice, self-help, and coping strategies and relaxation techniques. Websites were assigned 1 point for each tool (range 0-14).

##### Support for Mothers

Support for mothers quantified the support websites offered that were guided (or monitored) by an HCP. This included online and offline support, including message-based counseling, helplines, and group meetings. Again, 1 point was assigned for each resource available (range 0-12).

##### Additional Resources

Additional resources scored any other resources that might be useful to mothers, including links to external sites, audio-visual resources, book reviews, and leaflets (range 0-11). The scoring criteria for each category were based on Moore and Ayers [22]. However, any additional resources that were identified as part of the review were added to the scoring criteria *pro re nata*.

#### Website Quality

The quality of each website was examined using 9 subscales, each scored on a scale of 0-2 (equating to poor, mediocre, and good).

##### Contactability

Websites scored points if (1) the author was identified and (2) the contact information was provided.

##### Up-to-Date

Points were assigned if (1) there was evidence of regular website maintenance and (2) all of their links were functioning correctly.

##### Navigation

Websites with a clear menu or index that linked to all pages on the site were assigned 2 points. Websites that were relatively easy to navigate but needed several clicks to access all pages scored 1. If sites were difficult to navigate, they scored 0. Common reasons for scoring 0 were the lack of menu or index, the presence of many confusing or hidden links, a structure that causes users to get stuck in a navigation loop, or sites that necessitated the use of a search option to find relevant information.

##### Presentation

Websites that looked “clean,” with clear, uncluttered pages, with a good balance between text and pictures were assigned 2 points. Conversely, websites scored 0 if they were confusing and overcrowded, with too much information on a page and no pictures. A score of 1 was given to sites that fell between the 2.

##### Advertisements

Advertisements can cause users to have a negative experience of a website, distracting them from the main purpose of the site, and disrupting its presentation and usability. The maximum points were assigned to sites without any advertisements, 1 point was given to sites that had some advertising, but which was relatively inconspicuous, and 0 was assigned to sites that contained adverts that impaired user experience.

##### Accessibility

Websites that required fees or special software to access information were assigned 0 points; those that required users to create an account (for free) before they could access information were rated 1; and sites where the majority of information was freely available and easy to access scored 2.

##### Credibility

Websites scored points if they (1) included evidence-based content and (2) showed that information was legitimate by containing relevant references and citations. Information was deemed “evidence-based” if it included more than just anecdotal or personal opinion and had been previously identified in the literature as being associated with a PNA cohort. This information was often provided without including citations. As this type of information was often reported without the inclusion of references, an additional scoring criterion was added to capture information about the frequency of appropriate citations.

##### Engagement

Points were assigned for sites that (1) included information that was well-targeted or personalized for the audience (eg, that was presented in an easily relatable manner, eg, by couching symptoms and information in terms of real-life stories and experiences, and how they might manifest in this cohort) and (2) used methods designed to hold user interest (eg, presenting information in different formats or containing a degree of interactivity).

#### Audience Relationship

This section considered qualitative information about the websites’ relevance to a perinatal audience along 4 dimensions.

##### Website Specificity

This section classified whether the PNA information identified belonged to websites dedicated to PNA or whether the PNA information was just a subsection of a site dedicated to other topics.

##### Perinatal Anxiety Specificity

This involved specifying whether the information provided about PNA was done so in its own right, or whether it conflated PNA with perinatal depression.

##### Location

As the location of the Web-owner may directly influence the relevance of content and resources offered, each website was given a country code.

##### Author

Finally, as the nature and content (and even credibility) of a website is likely to be influenced by its authors, each website was coded as being authored by one of the following: health institute, charity, a woman who had recovered from PNA, researcher, therapist, and other.

#### Readability

Finally, the initial paragraph of each site was copied into Microsoft Word to establish its Flesch-Kincaid Grade Level [35]. This measure uses a formula including total words, sentences, and syllables to calculate the level of education someone is likely to need (in years) to easily read the text. It is a reliable measure that is frequently used to assess how difficult it is to understand health information, and previous research considered the first paragraph a good representation of the content of websites [22]. Health education experts advise information to have a reading level of ≥8.

## Results

### Website Identification

We screened 4000 hyperlinks for eligibility. Search engine results yielded 47 websites that met the inclusion criteria, 3025 were duplicates and 575 were excluded with the following reasons: they included <500 words on PNA (n=188), focused solely on other perinatal mental illnesses (n=9), general anxiety and childhood anxiety (n=37), or general mental health (n=21); they were news items (n=48), magazines (n=26), blogs (n=33), forums (n=7), Facebook groups and other social media (n=3), PDFs (n=11), videos (n=6), scholarly papers (n=87), training courses (n=17), paid online therapy (n=38), sales promotions (n=37), other search engine results (n=3), and broken links (n=4). Eligible websites were examined for hyperlinks to other websites (n=400). Resultant links were assessed for eligibility and yielded a further 3 websites for inclusion.

### Measure Reliability

In total, 50 websites were reviewed by the primary author. To ensure the reliability of ratings, 10% (5/50) of the websites were selected using an online random number generator [36] and reviewed independently by the second author. This method was chosen to mirror a similar review of PND-focused websites [22]; furthermore, calculating the interrater reliability on a small subsample of cases and generalizing results to the full sample is a common, acceptable method when time and resources do not allow double ratings for all cases [37]. Intraclass correlations (ICC) revealed an excellent degree of reliability for the subscales *perinatal anxiety information* (ICC=.95), *website quality* (ICC=.94), and *additional resources* (ICC=.96), while *treatment and screening* displayed good reliability (ICC=.75). Discrepancies predominantly arose from missed information resulting from poor site navigation.

### Accuracy of Information

**Figure 1:**
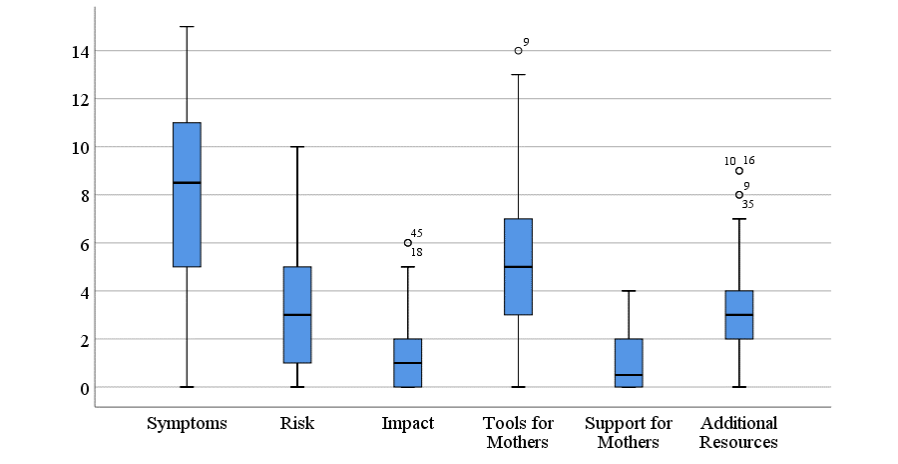
Distribution of scores obtained by the websites on the information and resources scales.

Figure 1 shows the distribution of scores for information on symptoms, risk factors, and impact given by websites. All but 2 websites referred to at least one symptom of PNA, although the number of symptoms reported by the sites was variable (range 0-15; mean 8.02 [SD 3.97]). None of the sites reported all 19 symptoms, and 20% (10/50) reported <5. Where symptoms were described, the vast majority tended to be related to anxiety symptoms observed in the general population (mean 5.60 [SD 2.86]), rather than PNA-specific symptoms (mean 1.68 [SD 1.25]). The most frequently mentioned symptoms can be seen in Table 1, alongside frequently reported risks and impact information. Confusingly, 20% (10/50) of the websites included some of the symptoms on the rating checklist but did so in relation to PND, and not PNA (these were not scored).

The information presented for risk factors was also variable (range 0-10; mean 3.16 [SD 2.53]). None of the sites reported all 11 risk factors, 98% (49/50) reported <7, and 20% (10/50)reported none.

Impact information occurred the least with 42% (21/50) of sites failing to report anything on this scale (range 0-6; mean 1.46 [SD 1.74]). While 60% (30/50) of sites listed one or more impacts on the mother (range 0-4; mean 1.18 [SD 1.24]), only 22% (11/50) mentioned infant outcomes (range 0-4; mean 0.48 [SD 1.01]).

The total score for information accuracy was created by summing symptoms, risk factors, and impact scores, with a possible range of 0-43. However, the actual range observed was 1-25 (mean 12.64 [SD 6.06]).

### Treatment and Screening

Most sites included information on treatment (46/50, 92%); 37 treatments were suggested with the most common being medication (38/50, 76%), cognitive behavioral therapy (28/50, 56%), and cognitive therapy (16/50, 32%).

In contrast, only 38% (19/50) of the sites contained mental health screening information. Ten scales were mentioned overall, with the EPDS cited most frequently (14/50, 28%). All other scales were generic mental health scales and not specific to the perinatal period. None appeared more than twice.

No sites contained inaccurate information or recommended alternative treatments over treatment from an HCP.

**Table 1:** The most commonly provided information across the websites.

Information provided		n (%)
**Symptoms**		
	Worry about infant safety and welfare	41 (82)
	Persistent unjustified worry	40 (80)
	Somatic symptoms of panic	36 (72)
	Obsessive and intrusive thoughts	70 (35)
**Risk factors**		
	Previous traumatic life event	30 (60)
	Negative birth experience	20 (40)
	Stress during pregnancy	20 (40)
	Poor social (or partner) support	19 (38)
**Impact**		
	Relationship issues or sexual dysfunction	13 (26)
	Difficulty fulfilling family roles	11 (22)
	Low birth weight	9 (18)
	Adverse effects on infant development	6 (12)

**Table 2:** The most commonly used tools, support, and resources across the websites.

Available help		n (%)
**Tools**		
	Information on how to seek help	41 (82)
	Standard self-help information	29 (58)
	Stigma reduction tools	22 (44)
	Stories from other mothers	22 (44)
**Support**		
	Telephone helplines	16 (32)
	Forums	5 (10)
	Referrals to health care professionals	5 (10)
	Therapy appointments	4 (8)
**Resources**		
	External links	27 (54)
	Associated social media	23 (46)
	Downloads	20 (40)
	Contacts	20 (40)

### Available Help

A range of help was provided across the sites including 14 different tools for mothers with PNA (see Multimedia Appendix 1 for full details). Of the sites evaluated, 98% (49/50) contained information about at least one tool (range 0-14; mean 5.36 [SD 3.39]). In addition, one site presented all 14 support tools on the measure (maternalmentalhealthnow.org).

In contrast, websites were relatively conservative in terms of the active support they offered, with only 50% (25/50) of sites offering some form of guided support (range 0-4; mean 0.94 [SD 1.20]). However, most provided links to additional resources (86% [43/50] contained at least one complementary resource; range 0-9; mean 3.20 [SD 2.41]). The most commonly supplied tools, support, and resources can be seen in Table 2. The total score for available help was calculated by adding together the 3 subscales (range 2-23; mean 9.50 [SD 5.75]).

**Figure 2:**
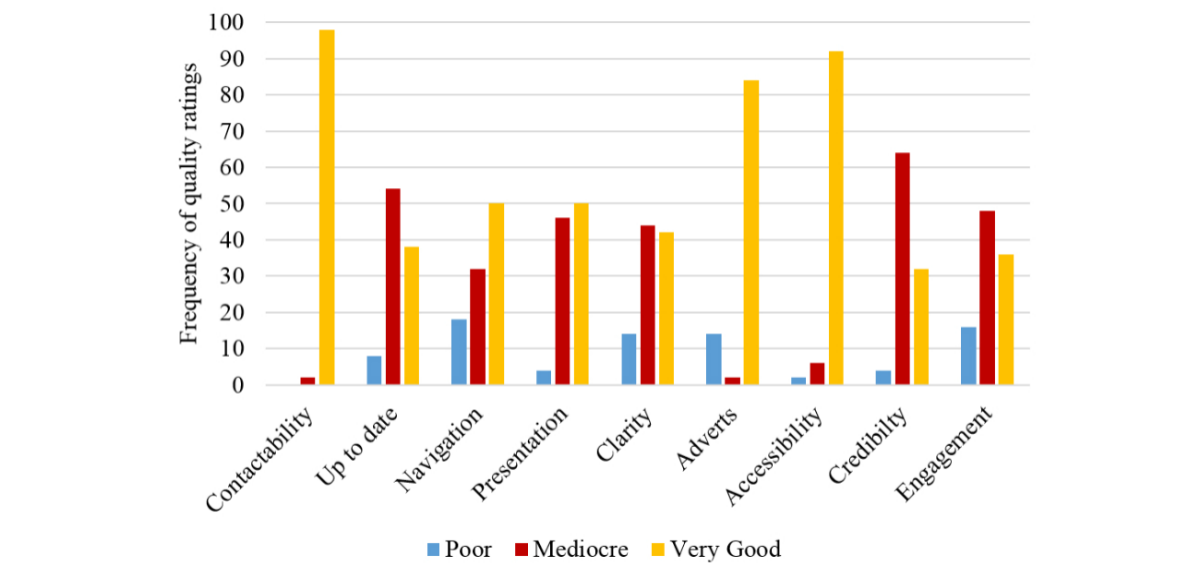
Variability of the website quality across different dimensions.

### Website Quality

The website quality varied substantially between the reviewed sites (range 6-17; mean 13.42 [SD 2.55]), although 60% (30/50) of sites scored over 13 (out of 18). Overall, the websites performed well on contactability (98%, 49/50), accessibility (92%, 46/50), and advertisements (84%, 42/50). Sites tended to do a bit worse on up-to-dateness and credibility, with the majority of sites scoring in the midrange on these dimensions (Figure 2). Sites were more likely to receive a “poor” rating on measures of navigation, clarity, advertisements, and engagement.

### Audience Relationship

An analysis of the website specificity revealed that 34% (17/50) of PNA information belonged to sites that were dedicated to perinatal mental health, especially the remaining 66% (33/50) were more general sites containing subsections (or pages) with information on PNA.

In addition, the PNA specificity was relatively low, with only 32% (16/50) of websites clearly separating PNA and PND. This proportion was similar for both sites dedicated to perinatal illness (5/17, 29%) and those covering a broader area (11/33, 33%).

Half of the websites were located in the United Kingdom, 10 in Australia, 9 in the United States, 5 in Canada, and 1 in New Zealand. Of sites whose ownership was transparent, 21 were created by a charity, 14 by a health institute, 4 by therapists, 3 by researchers, and 2 by women who had experienced PNA themselves.

Furthermore, it was noticed that websites tended to be aimed exclusively at mothers (24/50, 48%) or both mothers and HCPs (13/50, 26%). Only 3 sites were intended to be used solely by HCPs (3/50, 6%), and the remaining websites addressed different combinations of mothers, HCPs, and others (10/50, 20%).

### Reading Level

The reading level ranged from 7.1 to 37.4 and had a mean of 11.67 (SD 4.75). Only 16% (8/50) of sites had a reading level of ≥8 as recommended by health education experts [38,39].

### Top Websites

To be rated as a top website for HCPs and users, sites had to rank in the 75th percentile (or above) for information, website quality, and available help, and include accurate information about both screening and treatment. Only 4 sites met these criteria: (1) perinatal.anxietybc.com; (2) pada.nz; (3) halton.ca; and (4) mind.org.uk. However, websites (2) to (4) conflated perinatal anxiety and postnatal depression.

## Discussion

### Principal Findings

This study aimed to identify and review websites that contain information about PNA, evaluate the accuracy of information given, and the quantity and suitability of therapeutic advice and resources offered. This was done using an adapted version of Moore and Ayers’ [22] original measure, tailored to PNA. The quality of websites’ navigation, readability, presentation, and accessibility was also reviewed. An additional aim of this study was to identify the current most useful websites available for HCPs and their clients. Information was often inadequate and focused on symptoms rather than risk factors or impact on the mother and infant. In addition, websites often had information on treatment, but a few contained perinatal mental health screening information. While most sites provided at least some resources to support mothers, this was predominantly in the form of self-help or additional resources; active, guided support was infrequent. The website quality was extremely variable, with most presenting content that was difficult to read. The review suggests the top 4 websites for HCPs and their clients, and further advice is given to HCPs throughout this discussion.

This review identified 50 websites related to PNA, considerably fewer than 114 sites identified by Moore and Ayers in their review of PND websites [22]. This suggests PNA may still be comparatively underrecognized, underresourced, and underresearched [28,40]. No “gold standard” website was identified, as no single site contained complete information and resources, alongside a high score for the website quality. Mirroring the findings of Moore and Ayers [22], websites that scored well for information did not always score well for support and vice versa. However, 4 websites have been recommended for HCPs own use, and it is suggested they can recommend these sites to their clients considering the points raised in this discussion.

The information provided by websites was frequently incomplete, predominantly focusing on symptoms related to general anxiety rather than those that may be specifically related to PNA. This could prevent women relating to the information presented and prevent HCPs recognizing symptoms, thus potentially presenting a barrier to both help-seeking and treatment [15,41]. Conversely, while the most frequently mentioned symptom was “worry about infant safety and welfare,” sites rarely distinguished between common transient worries of this kind and those that are clinical, which may result in readers unnecessarily pathologizing normal behavior or experience. This is a factor HCPs may need to keep in mind when working with women in the perinatal period and when recommending these sites.

Websites often failed to deliver information on risk factors and impact. This could have negative repercussions for HCP users who need accurate information to help identify women at risk. Similarly, HCPs could point their clients to sites that have complete information on risk factors. Women might be assisted with preparing for prevention if they are informed of the risk factors and may be more likely to seek help if they are aware of the potential impact of untreated PNA on the mother and infant [22].

A key finding was that websites often conflated information on PNA with PND. This is concerning as women who access these sites may be looking to self-diagnose and may not identify with lists that contain both anxiety and depression symptoms (especially as depressive features tended to outweigh those related to anxiety). Although it is recognized that depression and anxiety can present together, this is not always the case [40]. Therefore, women who experience PNA in the absence of PND may conclude they do not have a mental health problem (and therefore not seek help) because their anxiety symptoms are not accurately represented by these websites. It is, therefore, noteworthy that only 1 of the 4 leading websites successfully separated PNA and PND. HCPs should, therefore, be cautious in recommending these sites and may need to provide their clients with supplementary evidence-based information that can help to separate these symptom profiles and relevant anxiety-related information.

Most websites mentioned treatment options, with pharmacological interventions being cited most often. While an effective treatment option for PNA, current research advocates nonpharmacological avenues as the first line of action [42]. Furthermore, pregnant and breastfeeding women may be reluctant to take medication in the perinatal period, so sites that fail to mention nonpharmacological options may put women off seeking help [43]. Positively, most websites did present alternatives to medication, with cognitive behavioral therapy and cognitive therapy most frequently suggested. As a recent review has shown these types of therapies to be effective for PNA [33], the inclusion of information about these treatment options is likely to be beneficial to both HCPs and women with PNA. HCPs should be aware that there is currently a dearth of research into efficacious PNA treatment and, thus, should ensure any treatment recommended by websites is supported by evidence.

In contrast to treatment information, screening tools were infrequently mentioned by the websites; when they were, the EPDS was most dominant. This raises some concerns, as the authors of the tool uphold that it does not measure anxiety [44], and other research suggests it does not reliably distinguish between anxiety and depression symptoms [45,46]. Thus, women self-screening might fail to recognize they have a problem, and HCPs might be ill-advised on the best measures for screening their clients. HCPs should consider providing clients with an alternative, validated PNA-specific measure such as the PASS [22], PSAS [23], and PRAQ-R [11].

All but one site provided mothers with access to at least one self-help tool, the most common being information on how to seek help, standard self-help advice, and stigma reduction. In addition, most sites presented additional resources that users could access. However, only half of the sites offered some form of active or guided support. This disparity is likely attributed to the challenges and cost implications involved in staffing and managing helplines, forums, support groups, etc. Whereas, additional resources (such as links, downloads, and generic social media pages) can be easily added to websites in a cost-effective and timely manner. However, it is worth noting that only half of the websites were based in the country where the review took place, which is likely to have serious implications for the accessibility and applicability of the tools, support, and resources offered. HCPs are, therefore, advised to check the availability of these resources in their clients’ locations before recommending the sites.

Overall, the website quality was found to be extremely variable. Sites were most likely to score poorly on navigation, clarity, advertisement, and engagement. In addition, and in line with previous research that has found online health information as difficult to read, most websites had a higher-than-recommended readability score [38,47]. These aspects are important to note, as they may prevent women from engaging with the sites, and getting the information they need. HCPs can use their discernment when recommending sites to tailor to individual needs.

In addition to the above recommendations made to HCPs, we also have some suggestions for future website development in this domain. Websites should include accurate and comprehensive evidence-based information that women can relate to, accompanied by high-quality supportive resources. In addition, these sites should be easy to use and read. Professionals developing PNA websites should be careful not to pathologize new parents’ concerns and instead recognize that some worries are common and dissipate over time. Equally, they should give some thought to the separation and identification of different anxiety disorders (eg, generalized anxiety disorder, childbirth-related worry, and other forms of anxiety such as obsessive-compulsive disorder), as their symptom profiles and treatment trajectories are likely to differ.

The website content would benefit from distinguishing between the symptoms of PNA and PND, but also note they can occur together. Other recommendations are that websites should present comprehensive risk factors and impact on the mother and infant to assist in the prevention and detection. Information on both pharmacological and nonpharmacological treatment options that are supported by evidence could avoid barriers to care. Future websites should consider including validated PNA-specific measures such as the PASS [27], PSAS [28], and PRAQ-R [14] to maximize their utility. Future research could investigate and develop self-help tools, thus enabling websites to provide resources that are cost-effective. The usability and readability could be improved by piloting sites with HCPs and women in the perinatal period.

### Limitations

One limitation of this study is that the measure used to assess websites is in its early stages of development and is yet to be validated. Currently, there is no validated measure that explicitly assesses the accuracy and appropriateness of information and resources provided, specifically related to PNA. This study builds on previous research that developed a rating scale specific to PND and aimed to develop a similar measure that focused on PNA. The measures were developed by both authors using DSM-V criteria, valid PNA scales, and evidence-based research. It is recommended that future works seek to validate the measure and compare it with other tools, such as DISCERN, which assess the quality of written health information [25].

Other limitations are that recommended websites are likely to date quickly with the evolving nature of the internet and growth in research and public awareness about PNA. Therefore, the top sites are likely to change over time. It is recommended that another review be done in the next few years to provide accurate top websites and include websites in all languages. A further limitation that needs to be addressed in future reviews is that the quality of the resources and efficacy of support tools provided were not established. Further research is also needed to explore how women use PNA websites and what they find most relatable and useful. It is likely that the number of PNA websites will expand and reviews might benefit from including a measure of the intended audience, for example, women in the community and those with more severe mental health needs. Appropriate resources might differ between groups and ratings should account for this. Overall, future reviews should recommend the top websites for information, resources, and website quality. The best websites should be clear in their focus on PNA information and resources and avoid confusing PNA and PND content.

### Conclusions

This review is the first to rate a substantial number of websites for information that was specific to PNA and available help for those experiencing it. The top 4 sites for HCPs and their clients are suggested. A key finding is that no website scored top for information, resources, and website quality. It is concerning that websites often conflated information about PNA with PND, as this could be misleading at best and at worst prevent women from seeking the help they need. To conclude, there is a need for websites to be developed that provide excellent evidence-based information that women can relate to and quality resources for women with PNA. These websites should clearly separate information on PNA and PND, be of sound quality for usability, easy to read, and built around research that identifies what women with PNA want from websites. This study provides guidance for HCPs recommending websites to their clients and professionals developing websites for PNA.

## Appendix

Multimedia Appendix 1 Rating scales for information and online resources.

## Abbreviations

DSM-V: Diagnostic and Statistical Manual of Mental Disorders, Fifth Edition
HCP: health care professional
ICC: intraclass correlations
PASS: Perinatal Anxiety Screening Scale
PNA: perinatal anxiety
PND: postnatal depression
PRAQ-R: Pregnancy-Related Anxiety Questionnaire-Revised
PSAS: Postpartum Specific Anxiety Scale
