# Supplementary material for: Advice for Health Care Professionals and Users: An Evaluation of Websites for Perinatal Anxiety
Source: JMIR Ment Health. 2018 Dec 20;5(4):e11464. doi: 10.2196/11464 (PMC6320400; doi:10.2196/11464)
Supplement: Multimedia Appendix 1 [file mental_v5i4e11464_app1.pdf]

Appendix 1 Rating scales for information and online resources

Information

**Symptoms**
Persistent, unjustified worry (general)
Persistent, unjustified worry about:<br>o Attachment/relationship with baby<br>o Maternal competence<br>o Infant safety and welfare<br>o Practical infant care<br>o Childbirth<br>o Psychosocial adjustment to motherhood<br>o Support as a mother<br>o Pregnancy/postpartum appearance
Irritability
Obsessive and intrusive negative thoughts
Checking behaviours (to neutralise negative thoughts)
Difficulty concentrating/ ‘brain fog’
Sleep disturbance and/or difficulty relaxing (unrelated to baby’s needs)
Mental symptoms of panic (e.g. feeling like something terrible is about to happen)
Somatic symptoms of panic (e.g. tension, palpitations, butterflies, hyperventilating, dizziness etc)
Agitation and restlessness
Avoidance of reminders of or talking about birth
Disturbing images, flashbacks or nightmares about birth
**Risk factors**
Pre-natal mood disorder (or previous experience of mental health issues)
1st time mother
Stress during pregnancy
Unplanned/unwanted pregnancy
Negative child birth experience
Infant born <27weeks, or admitted to the NICU
Previous loss of (or serious complications with) child/pregnancy
Poor maternal self-efficacy/ low self esteem
Poor social support/ relationship satisfaction
Previous traumatic (or highly stressful) life events (e.g. abuse, death/illness, redundancy, relationship breakdown etc…
Current/past substance use (smoking, drugs, alcohol)
**Impact**
Impact on the mother
Difficulty fulfilling family roles
Diminished responsiveness to infant cues
Attachment difficulties with their baby/other children
Fear of having another baby

Financial worries due to inability to work
Poor self-care, including following through with healthcare recommendations
Increased risk for future episodes of anxiety
Relationship issues/sexual dysfunction
Impact on the infant
Feeding problems/reduced breastfeeding duration
Increased fussiness and sleep problems (including colic)
Adverse effects on cognitive and social development
Lower birthweight
Emotional and conduct problems (but not associated with hyperactivity)

Available help

Mothers tools
Tests (e.g. Edinburgh Postnatal Depression Scale)
Myths about motherhood (dispelling unrealistic expectations of mothers)
Stigma surrounding PNI (i.e. confronting stigma and promoting help-seeking behaviour)
How to gain access to/understand medical notes
What to say to healthcare professionals (including letters)
How to seek help
Standard self-help (i.e. limited advice on nutrition, sleep, exercise and asking others for help)
Tips for family
Relaxation techniques
Coping strategies
How to think (e.g. advice on positive thinking)
PNI issues
Prevention
Stories
Mothers support
Forum
Email/letter
Chat
Personal messaging (a private message sent to another user in a forum or chat room)
Telephone
Group meetings
Home visits
Referrals
Social media support
App(s)
Additional resources
Links
Downloads
Audio/visual

Podcasts
Contacts
Articles/research
Book recommendations
Quotes/poems
Generic social media site (i.e. does not offer support)
Blogs
Newsletter/e-Bulletin
